# Exploring private sector perspectives on barriers and facilitators in availing tuberculosis care cascade services: a qualitative study from the Indian state

**DOI:** 10.1186/s12875-023-02244-w

**Published:** 2024-01-02

**Authors:** Harsh D Shah, Shalu Chaudhary, Bharat Desai, Jay Patel, Sandul Yasobant, Priya Bhavsar, Somen Saha, Anish K Sinha, Deepak Saxena, Yogesh Patel, Bhavesh Modi

**Affiliations:** 1 Department of Public Health Science, Indian Institute of Public Health Gandhinagar (IIPHG), Opp. Air Force Head Quarters, Nr. Lekawada, Gandhinagar, Gujarat 382042 India; 2 State Health System Resource Center, Government of Gujarat, Gujarat, India; 3 School of Epidemiology and Public Health, Jawaharlal Nehru Medical College, Datta Meghe Institute of Medical Sciences (Deemed to be University), Wardha, India; 4 John Snow India Pvt. Ltd. (JSIPL), New Delhi, India; 5 Department of Community & Family Medicine, All India Institute of Medical Sciences, Rajkot, Gujarat India

**Keywords:** Tuberculosis, Public Private Partnership, TB elimination, TB Care Cascade.

## Abstract

**Introduction:**

The private sector plays an important role in tuberculosis (TB) elimination by providing access to quality TB care services like diagnosis and treatment, advocacy for preventive measures, innovation to address challenges in TB elimination, vaccines etc. The study aims to understand the perspectives of private practitioners on patients’ TB care cascade to reinforce existing interventions by assuring the quality of care to TB patients.

**Methods:**

The study utilized a qualitative design through in-depth interviews of private practitioners and was conducted in Ranchi and Purbi Singhbhum District of Jharkhand State from March-August 2021. The pilot-tested, semi-structured, open-ended interview guide questionnaire collected information from private practitioners on various aspects of the TB care cascade. The data from the provider interviews were transcribed into multiple codes and themes on the TB program. An inductive analysis was carried out with a focus on content credibility to eliminate bias. Ethical approval was received from the Institutional Ethics Committee of the Indian Institute of Public Health Gandhinagar (IIPHG), India. Written consent was taken from the private practitioners involved in the study.

**Result:**

In-depth interviews of 17 private practitioners reveal various factors contributing to delays in TB care cascades, especially delay in access to TB diagnosis and TB Care, delay in providing treatment once after diagnosis and poor adherence to the TB treatment. According to the perception of private practitioners, there was an array of client, provider and system side factors affecting the TB care cascade gaps positively and negatively. Positive aspects mainly emerged from interviews: strong governance, consistent supply chain management, innovative PPP models and financial schemes reducing out-of-pocket expenditure (OOPE). Various factors affecting the TB care cascade negatively include awareness among the patient, socio-economic status, approach and decision-making power of providers, adverse effects of drugs, staff capacity building, etc.

**Conclusions:**

Engaging private practitioner in TB elimination efforts is critical to achieving global targets and reducing the burden of TB. The study helps to determine geography-specific barriers and facilitators of the TB care cascade to achieve the aim of providing universal access to TB healthcare with the inclusion of private practitioners.

## Background

India contributes roughly 26% of global tuberculosis (TB) burden. With 27% of the drug-resistant TB burden, India has the biggest number of drug-resistant TB patients in the world [1]. Hence, it is vital to accelerate TB prevention and care interventions under the National TB Elimination Program (NTEP). Under the program, the National Strategic Plan for India 2012–2017 calls for “Universal access for quality diagnosis and treatment for all TB patients in the community” [2]. The NTEP has set a new target to ensure ‘universal access’ to quality diagnosis and treatment for all TB patients in the community. One of the essential pillars towards obtaining universal access is to ‘expand efforts to engage all care providers, public and private [3].

More than half of the TB patients in India seek care from the private sector [4]. The private sector contributes around 32% of the TB notification rate out of the country’s total [5]. Despite several efforts made by NTEP, the involvement of the private sector is scattered across the country. Gaps in the TB care cascade include people with active TB not having access to diagnosis and support for completing treatment. This was observed more among patients diagnosed in the private sector than in the public sector. A comparative analysis of socio-demographic and clinical profiles revealed distinct characteristics between TB patients seeking care at private and public healthcare facilities. Private sector providers exhibited a higher propensity for utilizing chest X-rays for diagnostic purposes and administering unregulated and unmonitored treatment regimens. Notably, private sector providers accounted for over 60% of total TB drug sales. In contrast, public sector providers demonstrated a preference for sputum microscopy and adhered to regulated treatment protocols [6–12]. The private sector TB care cascade is marred by a multitude of challenges, including diagnostic disparities, suboptimal patient management, knowledge gaps, and a lack of standardized diagnostic and treatment protocols. These shortcomings collectively contribute to the compromised quality of TB care in the private sector [7, 8, 10–17].

There is a need to develop separate interventions for private practitioners and their TB patients because the problems faced within the public and private sectors are different. The study aimed to understand the perspectives of private sector experts on patients’ TB care cascade to reinforce existing interventions by assuring the quality of care to TB patients.

## Methods

### Study design

The cross-sectional qualitative research was carried out using a grounded theory approach and thematic analysis. In-depth interviews with respondents were chosen using purposive sample approaches to obtain data. For data analysis, content analysis was used.

### Study settings

The study was conducted in the Jharkhand state of India from March 2021 to August 2021. The state has 24 districts, which are grouped into five divisions. Out of 24 districts, two districts, namely, Ranchi and Purbi Singhbhum, were selected based on feasibility and convenience. Both districts were a part of the researchers’ project districts for various quality interventions across the NTEP in coordination and support with the state health department. Also, both districts have shown a private sector notification contribution of around 40% more than the rest of the districts as per the real-time TB information management system-Ni-kshay [18].

### Study participants and sampling

The study participants were private practitioners registered and notifying the TB patient to the health department through Ni-kshay portal or any communication channels. The list of the clinics/single private practitioners who had notified more than 50 TB patients in the previous two years were compiled from the portal and based on the respondents’ consent, number of active TB patients in consultations and having minimum MBBS (Bachelor of Medicine and Bachelor of Surgery) qualification, the purposive technique with the highest sampling variance was selected. In the study geography, 57 medical practitioners notified TB cases to the Government health department in the previous year. To ensure a diverse population relevant to the study, 17 participants out of 43 approached were interviewed over a 2-month data collection period. Among them, 12 were postgraduates, and five were general practitioners (MBBS).

### Study instruments & data collection

In-depth interviews were done in the regional language using an interview guide by the principal investigator and co-investigators trained in NTEP programme guidelines. The interview guide was meticulously crafted to elicit comprehensive insights into the experiences of private practitioners across the TB care cascade, encompassing both system-level and demand-side perspectives. The guide delved into various aspects of TB management, including practitioners’ views on government support, NTEP initiatives, diagnostic workflows, treatment modalities, public health actions, comorbidity management, follow-up procedures, and the challenges and solutions they encounter in their daily practice. Consent was acquired, and interviews were electronically recorded. The provider interview guidelines underwent a pilot testing among private practitioners in the Ranchi district of Jharkhand. The pilot testing revealed minimal alterations to the interview guide, with only 2–3 qualitative questions on treatment regimens and diagnostic workflows requiring modifications in response to practitioner feedback. Guided by a grounded theory methodological approach, questions were refined and adapted during concurrent analysis. The new theories or findings revealed during the interview was incorporated in an interview guide to identify the pattern of practices or responses from the study participants. The social cognitive theory was chosen as a guiding framework for this study since it aids in understanding human behaviours, intuitions, motivations, and processes of behaviour change that influence their response to relevant experiences.

The respondents picked the interview locations, and interviews were performed at a time that worked well for them. There were no follow-up interviews, and to ensure participant validation, the interview summary was discussed with the respondents. Once response repetition or consistency was noted, the team ceased interviewing respondents. Repeating incident descriptions for implementing the TB case notification method and related difficulties were classified as redundancy.

### Data analysis and management

The grounded theory of data collection was used to keep data collection consistent and to get respondent perspectives about TB patient management across the TB care cascade. The theory assisted us in conceptualising the hidden social patterns and difficulties associated with medical practices. The process of data collection, data analysis, and theory development was undertaken in an iterative process. A continual comparative analysis was used on the transcripts of the interviews, and inductive analysis was done with a focus on the content to uncover codes and themes [19, 20].

Data from provider interviews recorded on audio were verbatim transcribed and translated into English. The co-field investigators’ verbatim notes from the day of the interviews were used to generate the transcripts. The principal investigator (PI) read the transcripts to become familiar with the data once they were compiled. The data analysis strategy used was the open type of coding. Emergent codes from each respondent’s response were used in the analysis. The PIs read the sentences, determined their underlying meaning, and noted it in the margins by grouping related subjects, distinct topics, and other leftover topics. The PI identified themes for analysis when the procedure was finished. To create analytical categories, data pertinent to each category was found and looked at using a technique called constant comparison. A second researcher skilled in qualitative research techniques reviewed it to check for credibility and eliminate bias. Theme generation and coding convention selection were made by consensus and any disagreement was settled through conversation [21, 22]. The themes around the client, provider and system side were identified at stages TB care cascade services and codes were mapped to identify the factors related to the delays in services.

## Results

In-depth interviews with 17 private doctors were conducted to discover their views regarding various factors causing delays in the TB care cascade. The significant factors that emerged were either contributing to delay in accessing TB Care (A) and diagnostic services (B), delay in treatment initiation after diagnosis (C) or causing poor treatment adherence (D). Emerging factors influencing each cascade component were categorized under client-related, provider-related, or system-related sections. These factors, along with the corresponding numbers of responses, were documented in the tables.

### The delay during the first phase: delay in availing of TB care

- The majority of the factors causing a delay in accessing TB care were perceived to be from the client’s side. A few aspects related to the providers, or the system caused the delay in accessing TB care. (Table 1)
- According to the perception of the private practitioners, the health-seeking behaviour of the patients, which was also influenced by the socio-economic status of the patients, was a significant factor leading to delays in accessing TB care.

**Table 1:** Factors related to availing TB consultation and care services

Client Factors	• Awareness about the disease (5) • Health-seeking behaviour and socio-economic status (4) • Disease Presentation (6)
Provider Factors	Informal & Unqualified Providers (8)
System Factors	Need for robust Active Case Finding (2)

*“This is a rural area and, in this area, educated and less educated people are there. Many people have misinformation regarding TB especially among people with less health education. Initially, when symptoms develop, patients are not visiting doctors due to poor health-seeking behaviour. So, this is the reason for the late consultation. When severe symptoms develop, then they come for consultation.“-* (A Pulmonologist and Ex. Govt. official).

*“Health is not a priority of many patients, especially in Jharkhand and Bihar. People are ready for everything (shaadi ke liye paise rakhte he) [money for weddings is kept aside] but they are not caring about health.“-* (A Physician).

- To address this delay, strengthening the active case finding of TB, along with targeted and effective awareness activities in the community regarding all the aspects of TB and the services provided by the government, has to be the strategic mainstay to address the delay in access to TB care.

*“We saw a patient yesterday and we were regretting a lot. He was suffering from cough since past three months and was detected TB Positive…came from far away. When we come across such type of patients, we panic a lot as we have developed and providing many things, yet we find such a patient who is suffering from cough for three months and is now coming for treatment and this type of the delay affects the outcome. We should focus on survey and follow up through existing grass root workers on continuous bases. The system of the DOTS centre alone is not enough.“-* (A Pulmonologist).

- Another factor which caused the delay in accessing TB care was the pathological presentation of the clients. In the case of Extrapulmonary TB (EPTB), the presentation is often symptomless, diagnosed clinically or radiologically coincidentally during procedures undertaken for other case management.

*“We diagnosed TB cases coincidentally during the laparoscopic procedure being carried out in our IVF centres. The majority of the patients were of genital tuberculosis.” -* (A Gynaecologist).

- In the case of the classical presentation of TB, a recurrent emerging factor was the presence of informal and untrained providers in the state. Clients’ poor health-seeking behaviours, which are frequently a result of their low socio-economic level, poor health literacy, and the existence of informal providers whom the client chooses to contact, cause delays in receiving prompt and effective TB care.

*Many cases presented very late due to quack consultation. Sometimes it took 3 to 4 month and during that patients become very severely ill after that they refer patient to the private doctor.* (A Physician)

*I have found prescriptions...I have seen people saying they had consulted the practitioner in that area who did not have a degree….and that may be a reason for the delayed presentation.* (A Pulmonologist)

### Time lost in identifying the disease: delay in accessing TB testing

- When accessing the diagnostics for TB, the provider and system side factors affected the delay more than the client-side factors. The factors which emerged were primarily related to delays in timely access to appropriate diagnostic tests, either due to unavailability of the diagnostic modality or inappropriate diagnostic choice. (Table 2)
- A significant factor causing the diagnostic delay in the care cascade of TB was the unavailability of complete diagnostic tests in private and public setups. Even if the tests were available, the cost of the diagnostic tests in the private sector was another deterrent, which influenced the private providers’ decision to choose a diagnostic test, considering the clients’ economic status, which subsequently also affected their choice of the line of treatment.

**Table 2:** Factors related to access to diagnostic services

Client Factors	Socio-economic status (2)
Provider Factors	• Provider approach and decision (16) • Provider Capacity (10)
System Factors	• Service Delivery-Diagnostics-Non-availability (9) • Financing- Cost of test/ Affordability (3) • Human Resource-Capacity (2) • Service Delivery-PPSA & PPP models [Positive effect] (2) • Governance - Policy of free diagnostic provision [ Positive effect] (3)

*“Non-availability of culture and DST in government hospitals is a major problem.”* - (A Pulmonologist).

“*Non-availability of the second line investigation i.e., LPA (Line Probe Assay). Only after LPA, we should decide whether the patient need a longer regimen or a shorter regimen. In our hospital, we do LPA of our employees through an external outstate agency…But among non-employed patients, we are starting shorter regimens without knowing the injectable drug resistance status from the DOTS centre. We should ensure the LPA at least before starting treatment.“-* (A Pulmonologist).

*“Sometimes very poor patients do not afford the various investigations to be carried out for EPTB so we start treatment based on some investigations which they can afford.”* - (A Physician).

- On the other hand, the diagnostic services for nucleic acid amplification test (NAAT) were readily available in government setup. They were free of cost, and the private providers considered it to be very beneficial for the patients and in reducing the diagnostic delay in the care cascade.

*“Some costly tests (NAAT) are being carried out on free also they (government hospitals) are providing free medicines. This is very beneficial for the patients*.“- (A Physician).

- Another positive factor, according to the private providers, which reduced the diagnostic delay was the private sector engagement initiative through Patient Provider Support Agency (PPSA) “ALERT India” in Jharkhand State. This engagement was considered to be very beneficial for reducing the diagnostic delay and the out-of-pocket expenditure for patients seeking care from the private sector.

*“The new system (ALERTS) of transporting sample for CBNAAT made my job easier. Initially we are getting done CBNAAT from private lab which was very costly.“-* (A Pulmonologist).

- Missed diagnosis or wrong choice of diagnostics causing delay and ultimately worsening the case was a recurrent theme. However, only the availability of the diagnostics was not perceived to be enough to reduce the delays. Another major factor which emerged as a cause of diagnostic delay due to inappropriate diagnostic choice, was poor provider capacity. This was more so in the case of EPTB, where private practitioners perceived that a larger number of doctors, including specialists other than pulmonologists, have a lower capacity to diagnose EPTB. Frequent CMEs, targeted webinars and training programmes for all doctors involved in TB care emerged as a probable solution.

*“Patient didn’t have delayed presentation. The patient presented to one or other health practitioners who could not diagnose the patient leading to delay. I discharged a lady today. She is just a 23-year-old B tech student. She consulted a reputed hospital across one month without any diagnosis and brought to us in a very bad condition and we diagnosed tuberculosis.“-* (A Pulmonologist and Ex. Govt. official).

*"We need more training programs here because I found many missed diagnosed advanced cases, which means there is some gap in the training of non-pulmonologist practitioners. TB patients go to both private and government setups. I get patients here who were not satisfied with medical college and sadar also*"- (A Physician)

### A delay equivalent to negligence: delay in Initiation of TB Treatment following TB diagnosis

- The clients who undergo diagnostic tests and are confirmed as cases of TB arrive at a crucial stage in the TB care cascade as the cascade ahead not only determines their outcome but influences the disease burden of the community. According to the private providers’ perception, the factors that affected the delay in initiating TB treatment were mainly related to the client and provider’s behaviour and approach. (Table 3)
- A recurrent emerging factor causing the delay in initiating TB treatment was the stigma associated with TB, which led to a denial of the diagnostic results and, subsequently, rejection of the treatment from the TB patients and their families. The solution to this was perceived to be patient counselling and support from the provider’s side.

**Table 3:** Factors related to the initiation of treatment followed by confirmation of disease

Client Factors	• Awareness and stigma (8) • Client’s trust in the health system (3)
Provider Factors	Provider approach and decision (5)
System Factors	Drug Availability (2)

*“Stigma is there among patient…Hum to kahin jaate nahi hai to kaise huva, hamare family mein to kisiko bhi nahi he, hum to isike sath uthte bethte nahi he, corona ke chalte ham to kahi bahar nahi gaye to ham ko kese ho gaya? hamko TB ho gaya hamara kya hoga?)( We don’t go anywhere then how did we get it, nobody in our family has it, we don’t meet other people, due to Corona we haven’t gone anywhere then how did it happen? I have TB, what will happen to me now.) …We should counsel them regarding that.”* – (A Pulmonologist).

*“Still patients are not accepting the TB if they come positive. Still, they think that if anyone has TB, they have to live separately and eat separately. So, it takes time to counsel them regarding diet, lifestyle and regular medicine.”* – (A Gynaecologist).

- Hand in hand with stigma, poor awareness regarding TB also influenced the client’s approach towards accepting the diagnosis and the treatment. This was more in the case of EPTB due to the absence of classical symptoms of pulmonary TB.

“*People are not accepting EPTB…Madam hame khanshi nahi heto kese ho sakta hai? (Madam, I don’t have cough then how can it happen to me?) In such patients we have to counsel them”* – (A Gynaecologist).

- The client’s faith in the system was another barrier to starting therapy following diagnosis. Private healthcare providers claim that patients’ decisions to seek treatment were also impacted by their lack of confidence in the effectiveness of government-provided medications.

*“Somewhere insecurity about government medicine is observed among some patients…pata nahi kaam karega ya nahi (don’t know whether this will work or not)”* – (A Pulmonologist).

- The providers’ strategy and clinical judgement also affected the delay. In the case of drug-resistant TB (DRTB), the private providers would prefer to refer the patient to a government facility rather than treat them. The absence of MDR TB medications turned out to be a factor in the private provider’s decision.

*“I do not treat DRTB. I immediately refer DRTB patient to the government hospital. I treat DSTB only”. -* (A Physician and Ex. Govt. official).

*Problem tab hota hai jab MDR hota hai. (Problem happens when MDR happens) We don’t have all MDR medicines, also not available outside (private sector). Shortage of MDR drugs like clofazamine, ethionamide, PAS etc. Even sometime government also doesn’t have these.* – (A Pulmonologist).

### The critical last steps: ensuring treatment adherence & completion following treatment initiation

- After the initiation of medication, completion of the therapy course is of utmost importance as it has many consequences on patient and public health outcomes. According to the perception of the private providers, there were an array of client, provider and system factors which affected patients’ treatment adherence in a positive or negative manner. (Table 4)
- The socio-economic status of the patients was one of the major characteristics that was thought to be a contributing factor to poor treatment adherence by the private providers. Low-income clients who were geographically dispersed and encountered financial hardship when travelling for follow-up sessions would eventually discontinue once they felt better.

**Table 4:** Factors related to treatment adherence and outcome

Client Factors	• Socio-economic status (5) • Approach -discontinuation of medication on symptomatic relief (8) • Disease Presentation-Comorbidity (2) • Health Seeking Behaviour [Positive effect] (11)
Provider Factors	• Approach and decision (5) • Client-centric approach [Positive effect] (11)
System Factors	• Drug Availability (3) • Drugs- Consistent Supply Chain [Positive Effect] (10) • Drug Adverse effects (8) • Financing-Irregularities in receiving benefits of Nikshay Poshan Yojana (5) • Financing- Nikshay Poshan Yojana [Positive Effect] (6) • Service Delivery-PPSA [Positive Effect] (7) • Governance- Policy of free diagnostic provision [ Positive effect] (7)

*“Lack of money for transport and feeling well after 2 month of treatment is the reason for drop-out. Still financial problem is major issue.”* – (A Physician).

- However, a causal factor behind this also emerged as an independent factor, i.e. the client’s awareness about course of treatment and approach. According to the private providers, once the symptoms resolved and the clients started feeling better, they did not see the need to continue the medication and would eventually discontinue.

*“Many patients stopped medicine after one month when they feel good*.“- (A physician).

- Comorbidity was another factor that was identified. Clients experienced discomfort due to comorbid conditions or adverse drug interactions, making it difficult for them to follow up. (Lost to follow up)

*“Due to comorbidities and side effect also, patients left out. There was a lady with severe diabetes and arthritis, she developed joint pain also. So, she quit the medicine and we are unable to find her”- (*A Physician).

- Private practitioners claim that a key element in patient adherence and treatment completion is the clients’ positive attitude, which is driven by their awareness of the disease and influences how they seek health care. However, this factor also developed another side. Because they sought treatment from private institutions, the patients, in the opinion of the private providers, adhered to the course of therapy.

*“Awareness regarding follow up even after completion of treatment is good enough among most of my patients.“-* (A Physician).

*"Few patients are very sincere regarding follow up as this is private hospital."-* (A Gynaecologist)

- Not just the positive approach of clients, but the client-centric approach of the providers of repetitive counselling, sensitising about possible side-effects and diligently following up with the patients was also a driver of treatment completion.

*“My all patients had completed the full course. It depends on how much you follow up with the patients. We have an address and phone number, so the chance of default is very low.”* – (A Physician).

*“None my cases are at loss to follow up or default. We counsel them regarding possible side-effect in advance. So, if they comply for first one or two week, they complete the full course”* – (A Pulmonologist).

- However, from the interviews, it emerged that along with their client-centric approach, private practitioners also preferred to follow their clinical acumen instead of the programmatic guidelines.

*"We follow the guidelines but sometimes we need to do modification in regimen as per the requirement"-* (A Physician)

*"Sometimes, many practitioners in private sector prescribing TB regimen different than the existing guideline...jaise ki Fluroquinolone add kar diya. (E.g., adding Fluoroquinolone) Thus sensitisation of practitioners is needed for the same."* – (A Pulmonologist.)

- Sometimes, the non-availability of single drugs in the private sector was one of the concerns raised by the private practitioners because they did not prefer fixed drug combinations, citing the fear of side effects and adverse drug reactions.

*"In private sector I have one issue since last three months. I generally use the combination of rifampicin and Isoniazid with ethambutol and pyrazinamide separately. In the last three month this combination of Rifampicin and Isoniazid was difficult to get available in private sector, so I had to switch them to 3 or 4 Fixed Drug Combinations, which I generally try to avoid."- (A Pulmonologist and Ex. Govt. official)*

*"Whatever side-effect I found was immediately after starting a treatment or during IP. The most common side effect was gastritis and the next most common was hepatitis for which my incriminate was pyrazinamide. So, in those patients, I had to stop pyrazinamide. In fact, some incidences have happened that I have sent the patients to the government hospital where they receive the 4FDC. Still, those patients who suffered hepatitis and I have to subtract pyrazinamide, so those patients shifted from government regimen to my regimen." – (A Physician)*

- The private providers have to be made aware of this through regular CMEs. In addition to CMEs, the private providers suggested developing a platform where experts from the public and private sectors could regularly gather to debate issues and find solutions.

*“We should create a local expert group consisting of clinical and programmatic experts from the private and public sectors. This can be a good platform to discuss our problems and get solutions from the government.” – (A Physician)*.

*"Government and private both should work coordinatively. We found good cooperation from the government, also private practitioners giving good cooperation to the government" – (A Physician)*

- Additionally, numerous system- and provider-related factors impacted the care cascade’s treatment adherence; most had a reciprocal relationship. The Ni-kshay Poshan Yojana (NPY) of the Indian government was one of these elements. According to the private providers, treatment adherence was positively impacted by NPY, although the opposite was also true. Loss of follow-up and treatment halt was caused by irregularities or non-payment of NPY benefits.

*"It is good practice. Through that money, patient can buy good food, protein powder. That is helpful."* – (A Physician)

*"Incentives are very useful especially for poor people and also helps in motivating them to continue medicines."* - (A Pulmonologist and Ex. Govt. official)

*"Some patients also stop taking medicine if they didn't get the money (NPY) regularly as they are dependent."* (A Physician)

- Aside from NPY, it was thought that the political will and initiative to give patients free anti-TB medications, even in the private sector, would benefit them and stop non-adherence and delays in the treatment cascade. Moreover, a consistent supply chain of drugs, free of cost drugs, was also considered a strong positive driving factor of the care cascade.

*"Government had provided the anti TB treatment (ATT) medicine at our hospital at free of cost for TB patients that is good initiative." (A Physician)*

*"No supply chain issue regarding getting free drugs from the government." – (A Physician)*

*"Problem of loss to follow up due to high cost of the medicine at private sector is solved due to availability of the free drug supply at private clinic through government." – (A Pulmonologist)*

- The private sector engagement initiative through Patient Provider Support Agency (PPSA) “ALERTS India” was also considered a positive factor in ensuring treatment adherence.

*"Additionally, this system (ALERTS) is also beneficial for treatment. Also, MDR drug like Bedaquiline is not available in the market. So, we prefer this PPP model for treating patients. I have hardly a few patients taking medicines from government." – (A Pulmonologist)*

*"In case of a patient not coming for follow-up, they are being contacted by ALERT's Field Officer every 15 days." - (A Physician)*

### Effects of barriers and facilitators on the TB care cascade were mapped out

**Fig. 1:**
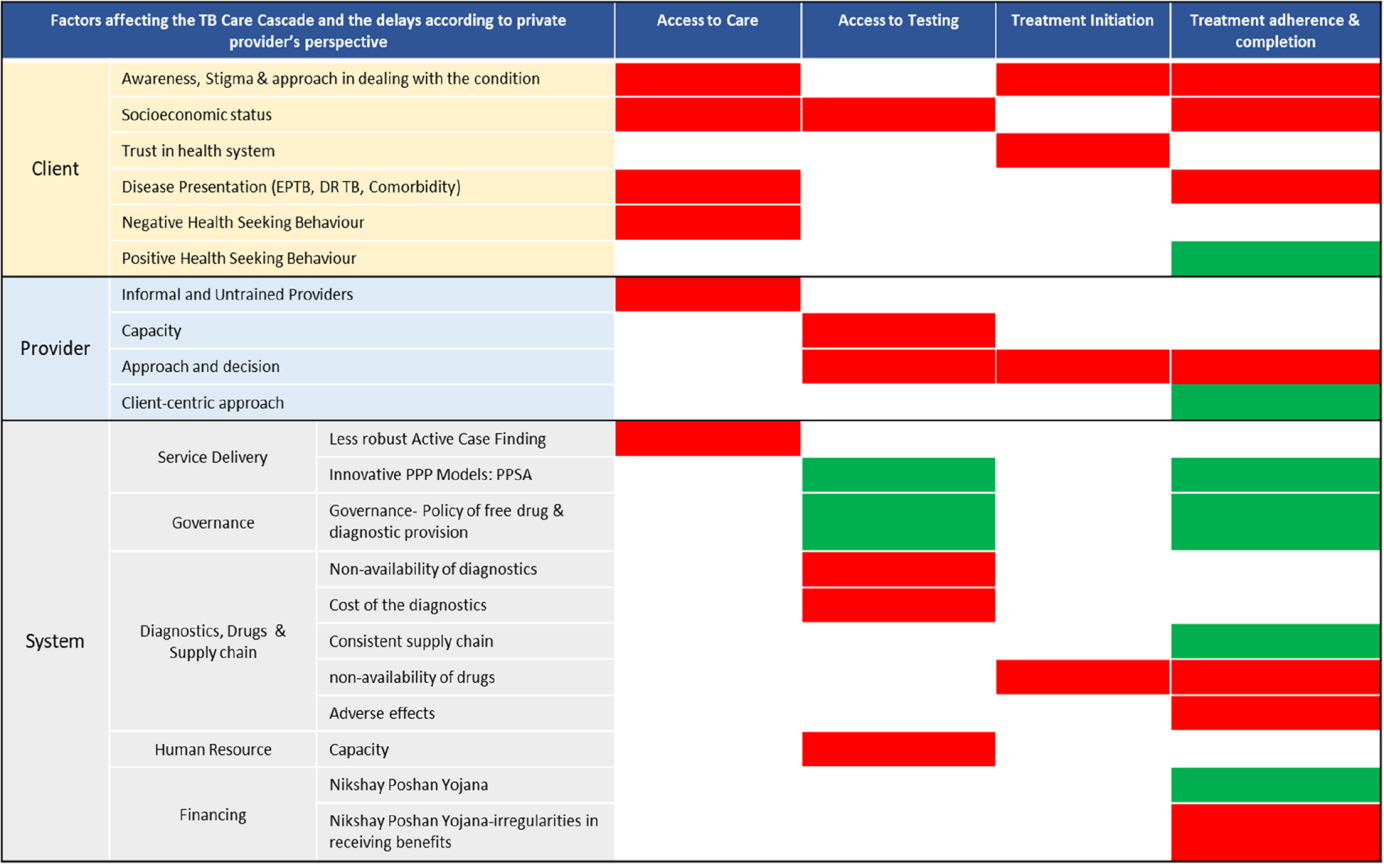
Factors affecting the TB care cascade. (Red: Negative effect, Green: Positive effect). EPTB = Extra Pulmonary TB, DR TB = Drug Resistant TB, PPP = Public Private Provide, PPSA = Patient Provider Support Agency

The interviews revealed the positive characteristics of system-side solutions, demonstrating the strong government commitment to eliminating TB (free drug and diagnostics, innovative service delivery, financing to reduce OOPE). The perspective suggested that the primary focus should be on increasing access to TB care by raising awareness, identifying cases early, initiating treatment as soon as possible after confirmation of the diagnosis, building provider capacity and adopting a client-centric strategy, and ensuring that all patients adhere to and finish their prescribed courses of treatment. (Fig. 1)

## Discussion

The National Strategic Plan 2012–2017 of NTEP advocates for creating and implementing engagement models to enhance and integrate private sector TB care within the programme [23]. The studies showed that the most significant risk factors for delay were the number and kind of initial providers consulted [13]. A considerable health system delay was observed by research on the postponement of TB diagnosis, and more than half of those with TB symptoms choose to start their search for care in the private sector. The behaviour factors of the providers (for TB patients) largely depend on the clients (TB patients) and so the treatment pathways will be different from patients of the public sector [24, 25]. There were numerous examples of public-private cooperation in the NTEP, and over time there has been a systemic increase in private-sector notification due to some of the successful demonstrations of private-sector engagement [26–29]. The behaviour factors of both should be addressed through the system in a more comprehensive way and creating a dialogue between all of three to resolve the gaps in TB care cascade services.

The nation needs to start taking the critical determinants of TB seriously, particularly literacy about TB and its management, comorbidity, and addictions, which have been connected to disease morbidity and related death. Addressing stigma and financial concerns requires collaboration across various sectors, involving community participation. The TB control program must also address the key systemic issues highlighted in the TB care cascade as contributing to the delay. These were a regular supply chain of diagnostic services and medications, monitoring of treatment adherence, private sector involvement, knowledge and information sharing platforms with the private sector and strategic actions to generate awareness and identify the TB cases from the community [15, 30, 31].

This study attempted to investigate the perception of the private sector in terms of experiencing delays along the care cascade for TB patients. By assessing the private providers’ perspective, we learned that many client, provider, and system factors had negative and positive effects on the care cascade. It was interesting to note that the approach and behavioural factors of the clients and the providers emerged the most and affected the care cascade at multiple points in either a negative or positive manner. The involvement of the private sector through the Patient Provider Support Agency (PPSA) intervention or by existing system should also serve as a platform to enhance knowledge on TB care among private practioners and their patients, collaborations to address the stigma by customised community participation channels, implement patient-centric support systems to ensure treatment adherence, and extend care services for comorbidities and disease related complications.

One of the limitations of this study is that the study involved only service providers of the selected districts of the state. The perspective of the private practitioners’ patients and service providers of public health facilities would have provided an in-depth understanding of the TB care cascade. This would have provided better insights for generalizability and transferability to other settings.

## Conclusion

Both system and demand side factors contribute uniquely, suggesting that the TB care cascade delay is multi-dimensional. A public-private mix model is well-known worldwide and significantly impacts TB control by facilitating patient access to TB services. Therefore, understanding the private sector specific barriers and facilitators of the TB care cascade is crucial if the nation is to provide universal access to TB care by including private-sector patients. A regular and institutionalised method should be set up for patients and providers with support system, which also aids in identifying care cascade gaps. The findings also suggested that NTEP program administrators pay attention to these different operational management components because some affect outcome indicators.

## Data Availability

The study was conducted with a qualitative approach with the grounded theory approach. The datasets used and/or analysed during the current study are available from the corresponding author on reasonable request. The datasets include codes and themes derived during the data collection and analysis phase.

## References

[1] Jeremiah C, Petersen E, Nantanda R, Mungai BN, Migliori GB, Amanullah F et al. The WHO Global Tuberculosis 2021 report – not so good news and turning the tide back to end TB. Int J Infect Dis. 2022;124 Suppl 1:S26–9. 10.1016/j.ijid.2022.03.011 PMC8934249 35321845

[2] Khaparde S. The national strategic plan for Tuberculosis step toward ending Tuberculosis by 2025. J Mahatma Gandhi Inst Med Sci. 2019;24(1):17.

[3] Sachdeva KS, Parmar M, Rao R, Chauhan S, Shah V, Pirabu R et al. Paradigm shift in efforts to end TB by 2025. Indian J Tuberculosis. 2020;67(4S):S48–60. 10.1016/j.ijtb.2020.11.001 33308672

[4] Arinaminpathy N, Batra D, Khaparde S, Vualnam T, Maheshwari N, Sharma L et al. The number of privately treated Tuberculosis cases in India: an estimation from drug sales data. Lancet Infect Dis. 2016;16(11):1255–60. 10.1016/S1473-3099(16)30259-6 PMC5067370 27568356

[5] INDIA TB REPORT. 2022. Coming Together to End TB Altogether. Ministry of Health and Family Welfare Government of India. [Internet]. 2022. Available from: https://tbcindia.gov.in/WriteReadData/IndiaTBReport2022/TBAnnaulReport2022.pdf.

[6] Kulshrestha N, Nair SA, Rade K, Moitra A, Diwan P, Khaparde SD. Public-private mix for TB care in India: Concept, evolution, progress. Indian J Tuberc. 2015;62(4):235–8. 10.1016/j.ijtb.2015.11.003 26970466

[7] Menberu M, Kar S, Ranjan Behera M. Review on public private mix TB control strategy in India. Indian J Tuberculosis. 2022;69(3):277–81. 10.1016/j.ijtb.2021.07.007 35760477

[8] Chopra KK, Arora VK. TB care in Private Sector: much more needed. Indian J Tuberc. 2016;63(4):217–18. 10.1016/j.ijtb.2016.12.001 27998491

[9] Ananthakrishnan R, D’Arcy Richardson M, van den Hof S, Rangaswamy R, Thiagesan R, Auguesteen S et al. Successfully engaging private providers to improve diagnosis, notification, and treatment of TB and drug-resistant TB: the EQUIP public-private model in Chennai, India. Glob Health Sci Pract. 2019;7(1):41–53. 10.9745/GHSP-D-18-00318 PMC6538134 30926737

[10] Daniels B, Shah D, Kwan AT, Das R, Das V, Puri V et al. Tuberculosis diagnosis and management in the public versus private sector: a standardised patients study in Mumbai, India. BMJ Glob Health. 2022;7(10):e009657. 10.1136/bmjgh-2022-009657 PMC9582305 36261230

[11] Stallworthy G, Dias HM, Pai M. Quality of Tuberculosis care in the private health sector. J Clin Tuberc Other Mycobact Dis. 2020;20:100171. 10.1016/j.jctube.2020.100171 PMC7332523 32642560

[12] Suseela RP, Shannawaz M. Engaging the Private Health Service Delivery Sector for TB Care in India—Miles to go! Trop Med Infect Dis. 2023;8(5):265. 10.3390/tropicalmed8050265 PMC10222514 37235313

[13] Sreeramareddy CT, Qin ZZ, Satyanarayana S, Subbaraman R, Pai M (2014). Delays in diagnosis and treatment of pulmonary Tuberculosis in India: a systematic review. Int J Tuberculosis Lung Disease.

[14] Das J, Kwan A, Daniels B, Satyanarayana S, Subbaraman R, Bergkvist S et al. Use of standardised patients to assess quality of Tuberculosis care: a pilot, cross-sectional study. Lancet Infect Dis. 2015;15(11):1305–13. 10.1016/S1473-3099(15)00077-8 PMC4633317 26268690

[15] Subbaraman R, Nathavitharana RR, Mayer KH, Satyanarayana S, Chadha VK, Arinaminpathy N et al. Constructing care cascades for active Tuberculosis: a strategy for program monitoring and identifying gaps in quality of care. PLoS Med. 2019;16(2):e1002754. 10.1371/journal.pmed.1002754 PMC6392267 30811385

[16] Yasobant S, Bhavsar P, Kalpana P, Memon F, Trivedi P, Saxena D. Contributing factors in the tuberculosis care cascade in india: A systematic literature review. Risk Manag Healthc Policy. 2021;14:3275–86. 10.2147/RMHP.S322143 PMC8364383 34408513

[17] Chijioke-Akaniro O, Onyemaechi S, Kuye J, Ubochioma E, Omoniyi A, Urhioke O et al. Challenges in engaging the private sector for Tuberculosis prevention and care in Nigeria: a mixed methods study. BMJ Open. 2023;13(9):e069123. 10.1136/bmjopen-2022-069123 PMC11148675 37709312

[18] Sunil Kumar. NIKSHAY- A Web-based Solution for Monitoring of TB Patients [Internet]. [cited 2022 May 27]. Available from: https://informatics.nic.in/uploads/pdfs/0f92f340_NIKSHAY.pdf.

[19] O’Brien BC, Harris IB, Beckman TJ, Reed DA, Cook DA. Standards for reporting qualitative research: a synthesis of recommendations. Acad Med. 2014;89(9):1245–51. 10.1097/ACM.0000000000000388 24979285

[20] Setia MS. Methodology series module 10: qualitative health research. Indian J Dermatol. 2017;62(4):367–70. 10.4103/ijd.IJD_290_17 PMC5527715 28794545

[21] Article R. Researcher as an instrument in qualitative research: challenges and opportunities. Adv Nurs Midwifery. 2016;25(90):27–37.

[22] Burla L, Knierim B, Barth J, Liewald K, Duetz M, Abel T. From text to codings: intercoder reliability assessment in qualitative content analysis. Nurs Res. 2008;57(2):113–7. 10.1097/01.NNR.0000313482.33917.7d 18347483

[23] Saha I, Paul B. Private sector involvement envisaged in the National Strategic Plan for Tuberculosis Elimination 2017–2025: can Tuberculosis Health Action Learning Initiative model act as a road map? Med J Armed Forces India. 2019;75(1):25–7. 10.1016/j.mjafi.2018.12.009 PMC6349649 30705474

[24] Anand T, Babu R, Jacob AG, Sagili K, Chadha SS. Enhancing the role of private practitioners in Tuberculosis prevention and care activities in India. Lung India. 2017;34(6):538–44 10.4103/0970-2113.217577 PMC5684812 29099000

[25] Satyanarayana S, Kwan A, Daniels B, Subbaraman R, McDowell A, Bergkvist S et al. Use of standardised patients to assess antibiotic dispensing for Tuberculosis by pharmacies in urban India: a cross-sectional study. Lancet Infect Dis. 2016;16(11):1261–68. 10.1016/S1473-3099(16)30215-8 PMC5067371 27568359

[26] Dewan PK, Lal SS, Lonnroth K, Wares F, Uplekar M, Sahu S et al. Improving Tuberculosis control through public-private collaboration in India: literature review. BMJ. 2006;332(7541):574–8. 10.1136/bmj.38738.473252.7C PMC1397734 16467347

[27] Pantoja A, Lönnroth K, Lal SS, Chauhan LS, Uplekar M, Padma MR et al. Economic evaluation of public-private mix for Tuberculosis care and control, India. Part II. Cost and cost-effectiveness. Int J Tuberculosis Lung Disease. 2009;13(6):705–12. 19460245

[28] Chauhan LS. Public-private mix DOTS in India. Bull World Health Organ. 2007;85(5):399.

[29] Central TB, Division M of H and FWG of India. National TB Elimination Program Guidelines [Internet]. [cited 2022 May 28]. Available from: https://tbcindia.gov.in.

[30] Padayatchi N, Daftary A, Naidu N, Naidoo K, Pai M. Tuberculosis. Treatment failure, or failure to treat? Lessons from India and South Africa. BMJ Glob Health. 2019;4(1):e001097. 10.1136/bmjgh-2018-001097 PMC6357918 30805207

[31] Shewade HD, Kokane AM, Singh AR, Parmar M, Verma M, Desikan P et al. Provider reported barriers and solutions to improve testing among Tuberculosis patients ‘eligible for drug susceptibility test’: a qualitative study from programmatic setting in India. PLoS ONE. 2018;13(4):e0196162. 10.1371/journal.pone.0196162 PMC5909888 29677210

